# Chebyshev model arithmetic for factorable functions

**DOI:** 10.1007/s10898-016-0474-9

**Published:** 2016-10-12

**Authors:** Jai Rajyaguru, Mario E. Villanueva, Boris Houska, Benoît Chachuat

**Affiliations:** 1 Department of Chemical Engineering, Centre for Process Systems Engineering, Imperial College London, South Kensington Campus, London, SW7 2AZ UK; 2 School of Information Science and Technology, ShanghaiTech University, 319 Yueyang Road, Shanghai, 200031 China

**Keywords:** Global optimization, Factorable functions, Chebyshev models, Taylor models, Interval analysis, Convergence rate

## Abstract

This article presents an arithmetic for the computation of Chebyshev models for factorable functions and an analysis of their convergence properties. Similar to Taylor models, Chebyshev models consist of a pair of a multivariate polynomial approximating the factorable function and an interval remainder term bounding the actual gap with this polynomial approximant. Propagation rules and local convergence bounds are established for the addition, multiplication and composition operations with Chebyshev models. The global convergence of this arithmetic as the polynomial expansion order increases is also discussed. A generic implementation of Chebyshev model arithmetic is available in the library MC++. It is shown through several numerical case studies that Chebyshev models provide tighter bounds than their Taylor model counterparts, but this comes at the price of extra computational burden.

## Introduction

Many complete search methods for problems in global optimization and constraint satisfaction hinge on the ability to compute enclosures for the range of nonconvex functions as well as sets defined by multiple equalities and/or inequalities [44]. A variety of parameterizations can be used to describe or approximate compact sets, including convex sets such as intervals, ellipsoids and polytopes as well as nonconvex sets for instance in the form of the image of a multivariate polynomial. Chachuat et al. [13] have argued that the foregoing parameterizations and many others can be cast as affine set-parameterizations with either convex or nonconvex basis sets. In particular, a number of arithmetics have been developed over the years that enable propagation of certain affine-set parameterizations through the atom operations of factorable functions in order to determine rigorous enclosures of their image sets. This includes interval arithmetic [40, 41], ellipsoidal calculus [28, 61], polyhedral relaxations [54, 55], and Taylor model arithmetic [11, 36], to name but a few. Other established techniques to relax sets or programs defined by nonconvex factorable functions involve constructing a pair of convex/concave bounds for each function, such as McCormick relaxations [38, 39] and $\alpha$BB/$\gamma$BB relaxations [1, 3, 23]. In the case of semialgebraic problems, hierarchies of linear matrix inequality (LMI) relaxations can also be constructed based on a semidefinite programming formulation of the sum of squares decomposition for multivariate polynomials [29, 46].

The focus in this paper is on *polynomial models* of multivariable functions, namely enclosures in the form of a polynomial approximant of given order and an interval remainder bound on the approximation error. Taylor models (or Taylor forms) are a special case of polynomial models, whereby the polynomial approximation matches the multivariate Taylor expansion of the function at a given point in the variable domain [43]. The idea of such interval polynomial enclosures dates back to Moore [40], and methods for computing the remainder bounds concurrently with the polynomial were later developed in the early 1980s [17, 51] and popularized from the mid 1990s by Berz et al. [7, 35, 36]. Similar in essence to interval arithmetic, a well-developed Taylor model arithmetic is available [8, 34], encompassing rules for binary sums, binary products and outer-compositions with a library of univariate functions such as $\exp (\cdot )$, $\log (\cdot )$ and $\sqrt{\cdot }$. For sufficiently smooth functions, the diameter of the remainder interval constructed with (*q*th-order) Taylor model arithmetic can be proved to be a high-order power ($q+1$) of the diameter of the variables domain [11]. This gives Taylor models a clear advantage over traditional interval extensions or centered forms for sufficiently narrow domains, but conversely it may result in a large overestimation or may even be poorer than naive interval evaluation over wider domains. Nevertheless, this approach has proved successful in computing tight enclosures for the solutions of differential equations and implicit algebraic equations [24, 33, 42, 49, 50, 53, 60], and it has enabled complete search for a range of global optimization or constraint satisfaction problems that could not be tackled using interval techniques alone (see, e.g., [4, 9, 31, 32, 47, 52]). Such higher-order inclusion techniques are indeed appealing in complete search applications based on branching or subdivision, where they can mitigate the clustering effect [15, 62].

A rather natural idea involves replacing the Taylor series approximation with a better one, ideally the minimax polynomial approximation, or near-minimax approximations such as Chebyshev series. The development of affine arithmetics [19] yielding first-order approximations can be seen as a precursor of this approach. Regarding univariate functions, improved variants based on the Chebyshev polynomials, Bernstein polynomials and others were widely used during the early 1980s under the name of ultra-arithmetic [26]. A comparison of univariate Taylor forms and Chebyshev forms in [27] showed that expansions in Chebyshev series may be orders of magnitude more accurate than expansions in Taylor series. The use of Chebyshev series expansion as an arithmetic is also the philosophy behind the package Chebfun by Trefethen et al. [6, 57], which has recently been extended to functions in two variables as well [56]. It should be noted that Chebfun relies on computations with functions to 15-digit accuracy, thus removing the need for propagating a remainder term, but the results are not validated per se. In contrast, Brisebarre and Joldeş [12, 25] developed an arithmetic for univariate functions based on Chebyshev interpolation polynomials and a remainder term. More recently, Dzetkulič [16] extended the previous work to enable enclosure of multivariate functions using truncated Chebyshev series and an interval remainder term, namely Chebyshev models. This latter work makes use of Chebyshev models as a means of propagating bounds for differential equations, but does not study the convergence properties of this arithmetic.

Building upon these existing contributions, this article presents a Chebyshev model arithmetic in order to compute enclosures for an inclusive class of factorable functions (Sect. 3). Besides formalizing the rules of addition, multiplication and composition as well as the polynomial range bounders, we describe an improvement of the remainder term in composition operations with certain univariate functions. We also present a convergence analysis of Chebyshev models, which investigates the propagation of local convergence properties through the addition, multiplication and composition operations (Sect. 4), and we discuss the global convergence of this arithmetic as the polynomial expansion order increases. Finally, we present numerical case studies illustrating the convergence properties of Chebyshev models and comparing their tightness and computational performance with Taylor models (Sect. 5). The underlying implementation of Chebyshev model arithmetic is part of the library MC++, which is made freely available at: http://omega-icl.bitbucket.org/mcpp/.

## Notation and background

The set of *n*-dimensional interval vectors is denoted by $\mathbb {IR}^{n}$. The midpoint and radius of an interval vector $X:=[x^{\mathrm{L}},x^{\mathrm{U}}]\in \mathbb {IR}^n$ are defined as ${{\mathrm{mid\,}}}X := \frac{1}{2} (x^{\mathrm {L}}+ x^{\mathrm {U}})\in \mathbb {R}^n$ and ${{\mathrm{rad\,}}}X := \frac{1}{2} (x^{\mathrm {U}}- x^{\mathrm {L}})\in \mathbb {R}^n$, respectively. The image of the set $X\in \mathbb {IR}^n$ under a continuous function $f:\mathbb {R}^n\rightarrow \mathbb {R}$ is given by

$$\begin{aligned} \bar{f}(X) := \{f(x)|x\in X\}\in \mathbb {IR}. \end{aligned}$$

The total variation of a (possibly discontinuous) function $g:\mathbb {R}\rightarrow \mathbb {R}$ on the interval $Y:=[y^{\mathrm {L}},y^{\mathrm {U}}]\in \mathbb {IR}$ is the quantity

$$\begin{aligned} \left. V[g]_Y := \sup \left\{ \sum _{i=1}^{N} \left| g(\xi _{i})-g(\xi _{i-1}) \right| \right| N>0,\quad y^{\mathrm {L}}=\xi _0<\xi _1<\cdots <\xi _N=y^{\mathrm {U}}\right\} , \end{aligned}$$

where $\xi _0,\ldots ,\xi _N$ is any finite partition of *Y*, and the supremum norm of *g* on *Y* is given by

$$\begin{aligned} \Vert g\Vert _Y := \sup \left\{ |g(\xi )||\xi \in Y \right\} \end{aligned}$$

Moreover, by a slight abuse of the notation, we use *V*[*g*] and $\Vert g\Vert$ when $Y=[-1,1]$.

Chebyshev polynomials of the first kind, denoted by $T_k$, are defined by the three-term recurrence relation

$$\begin{aligned} T_0(x) := 1, \quad T_1(x) := x, \quad T_{k+1}(x) := 2xT_k(x)-T_{k-1}(x). \end{aligned}$$

An explicit expression of these polynomials on $[-1,1]$ is

$$\begin{aligned} T_k (x) = \cos (k \arccos (x)). \end{aligned}$$

The Chebyshev polynomials are extremal polynomials with respect to the property that, for each $k>0$, $T_k(x)$ is the polynomial with the largest possible leading coefficient subject to the condition that its range is in $[-1,1]$ for $x\in [-1,1]$.

Any polynomial function $\mathcal {P}^q$ of degree *q* can be written in terms of the Chebyshev polynomials as [37]

$$\begin{aligned} \mathcal {P}^q(x) = \sum _{k=0}^{q} a_k T_k(x). \end{aligned}$$

An efficient way of evaluating such a linear combination of Chebyshev polynomials is using the Clenshaw algorithm [14],

$$\begin{aligned} \mathcal {P}^q(x) = a_0 + xb_1(x) - b_2(x), \end{aligned}$$

where the $b_k$’s are obtained using the (reverse) recurrence formula

1 $$\begin{aligned} b_k(x) = a_k + 2xb_{k+1}(x) - b_{k+2}(x)\,\quad \text {with}\quad b_{q+1}(x)=b_{q+2}(x)=0. \end{aligned}$$

Moreover, the product between two Chebyshev polynomials expands to

2 $$\begin{aligned} T_j (x)T_k (x)&= \frac{1}{2}\left( T_{j+k}(x)+T_{|j-k|}(x)\right) . \end{aligned}$$

The Chebyshev polynomials form a sequence of orthogonal polynomials with respect to the weight $\frac{1}{\sqrt{1-x^2}}$ on $[-1,1]$,

$$\begin{aligned} \int _{-1}^1 \frac{T_j(x)T_k(x)}{\sqrt{1-x^2}}dx = \left\{ \begin{array}{ll} \pi &{}\quad \text {if}\,j=k=0\\ \pi /2 &{}\quad \text {if }\, j=k>0\\ 0 &{}\quad \text {otherwise.}\end{array}\right. \end{aligned}$$

Any Lipschitz continuous function *f* on $[-1,1]$ has a unique representation as an absolutely and uniformly convergent series [58],3where the notation $\sum ^{\prime }$ indicates that the first term is halved. In general, the Chebyshev coefficients $a_k$ can be approximated using a numerical quadrature, possibly in combination with the fast Fourier cosine transform to improve the computation speed [21].

For practical purposes, a *q*th-order polynomial approximant $\mathcal {P}_f^q$ for *f* can be obtained from the partial sums of (3) as4The smoother the function *f* on $[-1,1]$, the faster the convergence of its approximants $\mathcal {P}_f^q$ as $q\rightarrow \infty$. The following bounds are available with regards to the accuracy of the partial sum $\mathcal {P}_f^q$, depending on the regularity of *f*:

- If *f* and its derivatives through $f^{(s-1)}$ for a given $s\ge 0$ are absolutely continuous on $[-1,1]$ and if $f^{(s)}$ has finite total variation, $V[f^{(s)}]<\infty$, then [58]
  5 $$\begin{aligned} \forall q\ge s+1, \quad \left\| f-\mathcal {P}_f^q\right\| \le \frac{2}{\pi }\frac{V[f^{(s)}]}{s(q-s)^s}. \end{aligned}$$
- If *f* and its derivatives through $f^{(q)}$ are continuously differentiable on $[-1,1]$, then [18]
  6 $$\begin{aligned} \left\| f-\mathcal {P}_f^q\right\| \le \frac{\Vert f^{(q+1)}\Vert }{2^{q}(q+1)!}. \end{aligned}$$

An alternative approach to obtaining a *q*th-order polynomial approximant is by constructing the polynomial interpolant $\widehat{\mathcal {P}}_f^q$ at Chebyshev points,7The polynomial $\widehat{\mathcal {P}}_f^q$ yields a near-minimax approximation, and counterparts to the error estimates in (5) and (6) are, respectively, [18, 58]

8 $$\begin{aligned} \forall q\ge s+1,\quad \left\| f-\widehat{\mathcal {P}}_f^q\right\|&\le \frac{4}{\pi }\frac{V[f^{(s)}]}{s(q-s)^s},\end{aligned}$$

9 $$\begin{aligned} \left\| f-\widehat{\mathcal {P}}_f^q\right\|&\le \frac{\Vert f^{(q+1)}\Vert }{2^{q}(q+1)!}. \end{aligned}$$

While the truncated Chebyshev series expansion $\mathcal {P}_f^q$ is generally a better approximation than the Chebyshev interpolation polynomial $\widehat{\mathcal {P}}_f^q$, the latter comes with the inherent advantage that the coefficients $\widehat{a}_k$ in (7) can be obtained from a few function evaluations only.

The (over-)approximation of multivariate functions using Chebyshev polynomials is at the heart of the present paper. Like in the univariate case, any *q*th-order polynomial function in *n* variables can be written in terms of the Chebyshev polynomials as

$$\begin{aligned} \mathcal {P}^q(x) = \sum _{|\kappa |\le q} a_\kappa T_\kappa (x), \end{aligned}$$

where $\kappa \in \mathbb {N}^n$ is a multi-index; the order of $\kappa$ is given by $|\kappa | := \sum \nolimits _{i=1}^n \kappa _i$; and $T_\kappa (x)$ is a shorthand notation for the expression $\prod \nolimits _{i=1}^n T_{\kappa _i}(x_i)$ for any $x\in \mathbb {R}^n$.

## Chebyshev model arithmetic

Similar to Taylor models [11, 43], Chebyshev models are estimators made by the sum of a multivariate polynomial in Chebyshev form and a remainder interval providing a bound on the approximation error. A formal definition follows:

### Definition 1

Let the function $f:Z\rightarrow \mathbb {R}$ be defined on $Z\subseteq \mathbb {R}^n$. For every interval vector $Y\subset Z$, let the multivariate polynomial $\mathcal {P}_{f,Y}^{q}:[-1,1]^n\rightarrow \mathbb {R}$ with $\mathcal {P}_{f,Y}^{q}(\xi ) := \sum \nolimits _{|\kappa |\le q} a_\kappa T_\kappa (\xi )$ and the symmetric interval $\mathcal {R}_{f,Y}^{q}\in \mathbb {IR}$ be such that

10 $$\begin{aligned} \forall \xi \in [-1,1]^n, \quad f({{\mathrm{mid\,}}}Y +\xi \circ {{\mathrm{rad\,}}}Y) - \mathcal {P}_{f,Y}^{q}({\xi }) \in \mathcal {R}_{f,Y}^{q}, \end{aligned}$$

where $\circ$ denotes the entrywise (or Hadamard) product. We call the pair $(\mathcal {P}_{f,Y}^{q},\mathcal {R}_{f,Y}^{q})$ a *qth-order Chebyshev model* of *f* on *Y*, and $(\mathcal {P}_{f,Y}^{q},\mathcal {R}_{f,Y}^{q})_{Y\subset Z}$ a *scheme of qth-order Chebyshev models* for *f* in *Z*.

Notice that, in the sense of Definition 1, the polynomial part $\mathcal {P}_{f,Y}^{q}$ of a Chebyshev model may be different from the actual truncated Chebyshev expansion of *f*. Notice also the need for scaling the variables in $[-1,1]$ for consistency with the definition and properties of Chebyshev polynomials of the first kind. The definition of schemes of Chebyshev models is in analogy with the scheme of estimators introduced by Bompadre et al. [10, 11] to support convergence analysis.

Similar in essence to interval (or Taylor model) arithmetic, our focus in the following subsections is on the development of an arithmetic that enables the construction of Chebyshev models for factorable functions, namely functions which can be represented by a finite number of binary sums, binary products and outer compositions with a univariate function. As well as the specification of these three “elementary” operations, developing such an arithmetic also calls for an initialization procedure for variables and constants, and the ability to bound the range of Chebyshev models.

### Initialization

Given a set $Z\subseteq \mathbb {R}^n$, each variable $z_i\in Z_i$ with $i\in \{1,\ldots ,n\}$ can be represented by a scheme of *q*th-order Chebyshev models $(\mathcal {P}_{z_i,Y}^{q},\mathcal {R}_{z_i,Y}^{q})_{Y\subset Z}$ with

11 $$\begin{aligned} \forall \xi \in [-1,1]^n,\quad \mathcal {P}_{z_i,Y}^{q}(\xi )&:= \left\{ \begin{array}{ll} {{\mathrm{mid\,}}}Y_i \, T_0(\xi _i) &{}\quad \text {if}\,q=0\\ {{\mathrm{mid\,}}}Y_i \, T_0(\xi _i) + {{\mathrm{rad\,}}}Y_i \, T_1(\xi _i)&{}\quad \text {if}\,q>0 \end{array}\right. \end{aligned}$$

12 $$\begin{aligned} \mathcal {R}_{z_i,Y}^{q}&:= \left\{ \begin{array}{ll} Y_i-{{\mathrm{mid\,}}}Y_i &{}\quad \text {if}\,q=0\\ {[}0,0] &{}\quad \text {if}\,q>0. \end{array}\right. \end{aligned}$$

Likewise, any real constant $r\in \mathbb {R}$ is trivially represented by the scheme of Chebyshev models $(r \, T_0,[0,0])_{Y\subset Z}$.

### Binary sum operations

Consider two functions $f_1,f_2:Z\subseteq \mathbb {R}^n\rightarrow \mathbb {R}$, and suppose that $(\mathcal {P}_{f_1,Y}^{q},\mathcal {R}_{f_1,Y}^{q})_{Y\subset Z}$ and $(\mathcal {P}_{f_2,Y}^{q},\mathcal {R}_{f_2,Y}^{q})_{Y\subset Z}$ are corresponding schemes of Chebyshev models. Then, a scheme of Chebyshev models $(\mathcal {P}_{f_1\pm f_2,Y}^{q},\mathcal {R}_{f_1\pm f_2,Y}^{q})_{Y\subset Z}$ for $f_1\pm f_2$ in *Z* is simply obtained by addition/subtraction of the polynomial parts and of the remainder parts,

13 $$\begin{aligned}&\displaystyle \forall \xi \in [-1,1]^n,\quad \mathcal {P}_{f_1\pm f_2,Y}^{q}(\xi ) := \mathcal {P}_{f_1,Y}^{q}(\xi ) \pm \mathcal {P}_{f_2,Y}^{q}(\xi ) \end{aligned}$$

14 $$\begin{aligned}&\displaystyle \mathcal {R}_{f_1\pm f_2,Y}^{q} := \mathcal {R}_{f_1,Y}^{q} \pm \mathcal {R}_{f_2,Y}^{q}. \end{aligned}$$

### Binary product operations

Consider again two functions $f_1,f_2:Z\subseteq \mathbb {R}^n\rightarrow \mathbb {R}$, and suppose that $(\mathcal {P}_{f_1,Y}^{q},\mathcal {R}_{f_1,Y}^{q})_{Y\subset Z}$ and $(\mathcal {P}_{f_2,Y}^{q},\mathcal {R}_{f_2,Y}^{q})_{Y\subset Z}$ are corresponding schemes of Chebyshev models, with

$$\begin{aligned} \mathcal {P}_{f_1,Y}^{q}(\xi ) := \sum _{|\kappa |\le q} a^q_{Y,\kappa } T_\kappa (\xi ) \quad \text {and} \quad \mathcal {P}_{f_2,Y}^{q}(\xi ) := \sum _{|\kappa |\le q} b^q_{Y,\kappa } T_\kappa (\xi ). \end{aligned}$$

In order to arrive at a valid scheme of *q*th-order Chebyshev models $(\mathcal {P}_{f_1 f_2,Y}^{q},\mathcal {R}_{f_1 f_2,Y}^{q})_{Y\subset Z}$ for the product function $f_1 f_2$ in *Z*, we start by expressing the product between the respective polynomial parts,

$$\begin{aligned} \mathcal {P}_{f_1,Y}^{q}(\xi ) \mathcal {P}_{f_2,Y}^{q}(\xi ) = \sum _{|\lambda |\le q} \sum _{|\mu |\le q} a^q_{Y,\lambda } b^q_{Y,\mu }\, T_{\lambda }(\xi )\, T_{\mu }(\xi ). \end{aligned}$$

A complication with polynomial multiplication in Chebyshev basis is that, according to the property (2), each product term $T_{\lambda }(\xi ) T_{\mu }(\xi )$ generates $2^{N(\lambda ,\mu )}$ terms with

$$\begin{aligned} N(\lambda ,\mu ) := \mathrm{card}\{ i\in \{1,\ldots ,n\} \mid \lambda _i\mu _i>0 \} \le n. \end{aligned}$$

See also Sect. 5 for further discussions and related implementation considerations.

Introducing the map $\mathbb {P}_q:\mathbb {N}^n\rightarrow \mathbb {N}^n\times \mathbb {N}^n$ given by

15 $$\begin{aligned} \mathbb {P}_q(\kappa ) := \left\{ (\lambda ,\mu )\in \mathbb {N}^{2n} \Big | \begin{array}{l} \forall i\in \{1,\ldots ,n\},\quad \lambda _i+\mu _i=\kappa _i\ \vee \ |\lambda _i-\mu _i|=\kappa _i\\ |\lambda |\le q,\quad |\mu |\le q \end{array}\right\} , \end{aligned}$$

it follows from (2) that

16 $$\begin{aligned} \mathcal {P}_{f_1,Y}^{q}(\xi ) \mathcal {P}_{f_2,Y}^{q}(\xi ) = \sum _{|\kappa |\le 2q} c^q_{Y,\kappa } T_{\kappa }(\xi ) \quad \text {with}\quad c^q_{Y,\kappa } := \sum _{(\lambda ,\mu )\in \mathbb {P}_q(\kappa )} \frac{a^q_{Y,\lambda } b^q_{Y,\mu }}{2^{N(\lambda ,\mu )}}. \end{aligned}$$

Observe that the resulting product polynomial is of order 2*q* and that the index sets $\mathbb {P}_q(\kappa )$ are non-empty for $|\kappa |\le 2q$. A simple way of defining the polynomial part $\mathcal {P}_{f_1 f_2,Y}^{q}$ in the resulting Chebyshev model is therefore by retaining all the terms of order no larger than *q* as

17 $$\begin{aligned} \forall \xi \in [-1,1]^n,\quad \mathcal {P}_{f_1 f_2,Y}^{q}(\xi ) := \sum _{|\kappa |\le q} c^q_{Y,\kappa } T_{\kappa }(\xi ). \end{aligned}$$

Then, the corresponding remainder term $\mathcal {R}_{f_1 f_2,Y}^{q}$ can be obtained by assembling four terms, namely the multiplication of the two remainders, the multiplication of each remainder with a bound on the other polynomial range, and a bound on all the terms of order larger than *q* from the product of the two polynomials,

18 $$\begin{aligned} \mathcal {R}_{f_1 f_2,Y}^{q}&:= \mathcal {R}_{f_1,Y}^{q} \mathcal {R}_{f_2,Y}^{q} + \mathcal {R}_{f_1,Y}^{q} \left[ \mathcal {P}_{f_2,Y}^{q}\right] + \left[ \mathcal {P}_{f_1,Y}^{q}\right] \mathcal {R}_{f_2,Y}^{q} \nonumber \\&\qquad + \sum _{q<|\kappa |\le 2q} \left| c^q_{Y,\kappa }\right| [-1,1]. \end{aligned}$$

The latter hinges on the ability to compute bounds on the polynomial parts of the Chebyshev model operands, denoted by $[\mathcal {P}_{f_1,Y}^{q}]\supseteq \{\mathcal {P}_{f_1,Y}^{q}(\xi ) \mid \xi \in [-1,1]^n\}$ and $[\mathcal {P}_{f_2,Y}^{q}]\supseteq \{\mathcal {P}_{f_2,Y}^{q}(\xi ) \mid \xi \in [-1,1]^n\}$ here. Such range bounders for Chebyshev models are discussed later on in Sect. 3.5.

An important aspect about polynomial multiplication in Chebyshev basis is that the computed *q*th-order polynomial $\mathcal {P}_{f_1 f_2,Y}^{q}$ in (17) will not correspond to the *q*th-order Chebyshev expansion of $f_1f_2$ in general, even when $\mathcal {P}_{f_1,Y}^{q}$ and $\mathcal {P}_{f_2,Y}^{q}$ are themselves *q*th-order Chebyshev expansion of $f_1$ and $f_2$, respectively. The reason behind this discrepancy is that the Chebyshev coefficients in the *q*th-order expansion of $f_1f_2$ depend on terms of order greater than *q* in the Chebyshev expansions of $f_1$ and $f_2$, which are missing here because of the truncation. Dzetkulič [16] argue that the resulting Chebyshev models nevertheless provide a tighter approximation than by multiplying $\mathcal {P}_{f_1,Y}^{q}$ and $\mathcal {P}_{f_2,Y}^{q}$ in monomial basis.

### Univariate outer-composition operations

Consider the functions $f:Z\subseteq \mathbb {R}^n\rightarrow \mathbb {R}$ and $F:X\subseteq \mathbb {R}\rightarrow \mathbb {R}$. Suppose that $(\mathcal {P}_{f,Y}^{q},\mathcal {R}_{f,Y}^{q})_{Y\subset Z}$ is a scheme of *q*th-order Chebyshev models for *f*, such that $B^q_{Y}:=[\mathcal {P}_{f,Y}^{q}]+\mathcal {R}_{f,Y}^{q} \subseteq X$ for all $Y\subseteq Z$. Let the scaling function $\varPhi ^q_Y:Y\rightarrow [-1,1]$ be given by

$$\begin{aligned} \varPhi ^q_Y(y) := \frac{f(y)-{{\mathrm{mid\,}}}B^q_{Y}}{{{\mathrm{rad\,}}}B^q_{Y}} \quad \left( :=0\quad \text { if}\,{{\mathrm{rad\,}}}B^q_Y=0\right) , \end{aligned}$$

and denote by $(\mathcal {P}_{\varPhi ,Y}^{q},\mathcal {R}_{\varPhi ,Y}^{q})_{Y\subset Z}$ the corresponding scheme of Chebyshev models for $\varPhi ^q_Y$,

19 $$\begin{aligned} \left( \mathcal {P}_{\varPhi ,Y}^{q},\mathcal {R}_{\varPhi ,Y}^{q}\right)&= \frac{(\mathcal {P}_{f,Y}^{q},\mathcal {R}_{f,Y}^{q})-{{\mathrm{mid\,}}}B^q_{Y}}{{{\mathrm{rad\,}}}B^q_{Y}} \quad \left( =(0,[0,0])\quad \text {if}\,{{\mathrm{rad\,}}}B^q_Y=0\right) , \end{aligned}$$

with $\mathcal {P}_{\varPhi ,Y}^{q}(0)=0$. Suppose also that a scheme $(\mathcal {P}_{F,W}^{q},\mathcal {R}_{F,W}^{q})_{W\subset X}$ of Chebyshev models for *F* is available, with

$$\begin{aligned} \forall \zeta \in [-1,1], \quad \mathcal {P}_{F,W}^{q}(\zeta ) := \sum _{k=0}^q \varphi ^q_{W,k}\, T_k(\zeta ). \end{aligned}$$

We will discuss the computation of such a scheme at the end of this subsection.

In order to construct a valid scheme of *q*th-order Chebyshev models $(\mathcal {P}_{F\circ f,Y}^{q},\mathcal {R}_{F\circ f,Y}^{q})_{Y\subset Z}$ for the composite function $F\circ f$ in *Z*, we start by applying the Clenshaw recurrence formula (1) in order to determine a scheme $(\mathcal {P}_{\mathcal {P}_F\circ \varPhi , Y}^{q},\mathcal {R}_{\mathcal {P}_F\circ \varPhi , Y}^{q})_{Y\subset Z}$ of Chebyshev models for the (parameterized) composite function $\mathcal {P}_{F,B^q_{Y}}^{q}\circ \varPhi ^q_Y$,

20 $$\begin{aligned} (\mathcal {P}_{\mathcal {P}_F\circ \varPhi , Y}^{q},\mathcal {R}_{\mathcal {P}_F\circ \varPhi ,Y}^{q})&:= \varphi ^q_{B^q_{Y},0} + \left( \mathcal {P}_{\varPhi ,Y}^{q},\mathcal {R}_{\varPhi ,Y}^{q}\right) \left( \mathcal {P}_{\beta _1,Y}^{q},\mathcal {R}_{\beta _1,Y}^{q}\right) \nonumber \\&\quad \quad -\left( \mathcal {P}_{\beta _2,Y}^{q},\mathcal {R}_{\beta _2,Y}^{q}\right) \end{aligned}$$

21 $$\begin{aligned} \text {with:}\qquad \left( \mathcal {P}_{\beta _k,Y}^{q},\mathcal {R}_{\beta _k,Y}^{q}\right)&= \varphi ^q_{B^q_{Y},k} + 2 \left( \mathcal {P}_{\varPhi ,Y}^{q},\mathcal {R}_{\varPhi ,Y}^{q}\right) \left( \mathcal {P}_{\beta _{k+1},Y}^{q},\mathcal {R}_{\beta _{k+1},Y}^{q}\right) \nonumber \\&\qquad -\left( \mathcal {P}_{\beta _{k+2},Y}^{q},\mathcal {R}_{\beta _{k+2},Y}^{q}\right) , \quad k=0,\ldots ,q\end{aligned}$$

22 $$\begin{aligned} \left( \mathcal {P}_{\beta _{q+1},Y}^{q},\mathcal {R}_{\beta _{q+1},Y}^{q}\right)&= \left( \mathcal {P}_{\beta _{q+2},Y}^{q},\mathcal {R}_{\beta _{q+2},Y}^{q}\right) = (0,[0,0]). \end{aligned}$$

Observe that this recurrence consists of binary sum and product operations only, for which rules have already been established in Sects. 3.2 and 3.3. Observe also that when ${{\mathrm{rad\,}}}B^q_Y=0$, which occurs if either *f* is constant on *Y* or ${{\mathrm{rad\,}}}Y=0$, we have

$$\begin{aligned} \left( \mathcal {P}_{\mathcal {P}_F\circ \varPhi , Y}^{q},\mathcal {R}_{\mathcal {P}_F\circ \varPhi ,Y}^{q}\right)&:= \left( \varphi ^q_{B^q_{Y},0}, [0,0]\right) . \end{aligned}$$

Finally, the polynomial and remainder parts of the desired Chebyshev models $(\mathcal {P}_{F\circ f,Y}^{q},\mathcal {R}_{F\circ f,Y}^{q})_{Y\subset Z}$ are given by

23 $$\begin{aligned}&\displaystyle \forall \xi \in [-1,1]^n,\quad \mathcal {P}_{F\circ f,Y}^{q}(\xi ) := \mathcal {P}_{\mathcal {P}_F\circ \varPhi ,Y}^{q}(\xi ) \end{aligned}$$

24 $$\begin{aligned}&\displaystyle \mathcal {R}_{F\circ f,Y}^{q} := \mathcal {R}_{\mathcal {P}_F\circ \varPhi ,Y}^{q} + \mathcal {R}_{F,B^q_{Y}}^{q}. \end{aligned}$$

In order to use this approach, one needs the ability to construct Chebyshev models $(\mathcal {P}_{F,W}^{q},\mathcal {R}_{F,W}^{q})$ corresponding to a given library of univariate functions, such as $F(x)=\exp (x)$, $\log (x)$, $\sqrt{x}$, $\frac{1}{x}$, $\sin (x)$, |*x*|, etc.

- A systematic way of doing this is by computing a truncated Chebyshev expansion of *F* of order *q* as 25 Then, if *F* is $q+1$-times continuously differentiable on *W*, the truncation error can be bounded as
  26 $$\begin{aligned} \mathcal {R}_{F,W}^{q} = \frac{\left\| F^{(q+1)}\right\| _W ({{\mathrm{rad\,}}}W)^{q+1}}{2^{q}(q+1)!} [-1,1]. \end{aligned}$$
  Otherwise, if *F* and its derivatives through $F^{(s-1)}$ are absolutely continuous on *W* and $F^{(s)}$ has finite total variation $V[F^{(s)}]_W<\infty$ with $s<q$, a bound on the truncation error is given by
  27 $$\begin{aligned} \mathcal {R}_{F,W}^{q} = \frac{2}{\pi }\frac{V\left[ F^{(s)}\right] _W ({{\mathrm{rad\,}}}W)^s}{s(q-s)^s} [-1,1]. \end{aligned}$$
- Alternatively, a Chebyshev interpolating polynomial of order *q* may be computed as 28 The error bound $\mathcal {R}_{F,W}^{q}$ in (26) remains valid for any $q+1$-times continuously differentiable function *F* on *W*. Otherwise, the following bound may be used instead of (27) if *F* and its derivatives through $F^{(s-1)}$ are absolutely continuous on *W* and $F^{(s)}$ has finite total variation $V[F^{(s)}]_W<\infty$ with $s<q$,
  29 $$\begin{aligned} \mathcal {R}_{F,W}^{q} = \frac{4}{\pi }\frac{V\left[ F^{(s)}\right] _W ({{\mathrm{rad\,}}}W)^s}{s(q-s)^s} [-1,1]. \end{aligned}$$

In comparison with their Taylor expansion counterparts for sufficiently smooth functions, we note that the width of the remainder is smaller by a factor of $2^q$. Moreover, the Chebyshev approach remains applicable for continuous, yet nonsmooth, functions. For instance, since the total variation of the derivative of |*x*| on the interval $[-\rho ,\rho ]$ is finite, equal to 2, a bound for the interpolation error based on (29) is $\mathcal {R}_{|\cdot |,[-\rho ,\rho ]}^{q} = [-\frac{8\rho }{\pi (q-1)},\frac{8\rho }{\pi (q-1)}]$, for any $q>1$.

In practice, the bounds $\mathcal {R}_{F,W}^{q}$ on the truncation error may be tightened further if *F* meets certain regularity and monotonicity conditions. Under these extra conditions, it can be established that the maximum absolute error between *F* and its polynomial approximant $\mathcal {P}_{F,W}^{q}$—as given by (25) or (28)—always occurs at one of the endpoints of *W*. This result is formalized in the following lemma:

#### Lemma 1

Let the function *F* have an analytic extension on the closed unit disk in the complex plane and have all its successive derivatives $F^{(k)}$, $k\ge 1$ either of the same or alternating sign in $[-1,1]$. Then, we have

$$\begin{aligned} \Vert F-\mathcal {P}_{F}^q\Vert&= \max \left\{ \left| F(-1)-\mathcal {P}_{F}^q(-1)\right| , \left| F(1)-\mathcal {P}_{F}^q(1)\right| \right\} ,\\ \text {and} \quad \Vert F-\widehat{\mathcal {P}}_{F}^q\Vert&= \max \left\{ \left| F(-1)-\widehat{\mathcal {P}}_{F}^q(-1)\right| , \left| F(1)-\widehat{\mathcal {P}}_{F}^q(1)\right| \right\} \end{aligned}$$

for any $q\ge 0$, with $\mathcal {P}_{F}^q$ and $\widehat{\mathcal {P}}_{F}^q$ given by (4) and (7), respectively.

#### Proof

See “Appendix 1”. $$\square $$

This method of calculating the remainder is particularly useful as functions satisfying the conditions of Lemma 1 include the exponential, logarithm, inverse and square-root functions on any finite interval in their domains of definition.

### Range bounding

A range bounder of the Chebyshev model $(\mathcal {P}_{f,Y}^{q},\mathcal {R}_{f,Y}^{q})$ of a function $f:Z\subseteq \mathbb {R}^n\rightarrow \mathbb {R}$ on $Y\subset Z$ is the set $[\mathcal {P}_{f,Y}^{q}]+\mathcal {R}_{f,Y}^{q}\in \mathbb {IR}$, with $[\mathcal {P}_{f,Y}^{q}] \supseteq \{\mathcal {P}^q_f(\xi ) \mid \xi \in [-1,1]^n\}$. Such bounders are needed for computing binary product and univariate operations in Chebyshev model arithmetic—see Sects. 3.3 and 3.4 above, thus directly affecting the tightness of the computed Chebyshev models. Unfortunately, exact range bounding for multivariate polynomials is NP-hard, so one has to resort to some sort of over-approximation in practice.

A number of methods exist for bounding the range of multivariate polynomials in monomial form [20, 30, 36, 43], which can be readily adapted to their Chebyshev counterparts. Apart from the naive bounding of each term separately,

30 $$\begin{aligned} \left[ \mathcal {P}_{f,Y}^{q}\right] = a^q_{Y,0} + \sum _{0<|\kappa |\le q} \left| a^q_{Y,\kappa }\right| [-1,1], \end{aligned}$$

which may produce weak bounds, this subsection presents adaptations of: (i) the methods in [33] that involves exact bounding of the first- and diagonal second-order terms; and (ii) the method of Bernstein.

#### Exact bounding of first- and diagonal second-order terms

We start by rewriting the polynomial part in the form

$$\begin{aligned} \mathcal {P}^q_{f,Y}({\xi })&= \sum _{j=1}^{n} \left[ a^q_{Y,\kappa ^{2,j}} T_2(\xi _j) + a^q_{Y,\kappa ^{1,j}} T_1(\xi _j)\right] + \mathcal {H}^q_{f,Y}(\xi ), \end{aligned}$$

where $\kappa ^{1,j}$ and $\kappa ^{2,j}$ are the multi-indices given by $\kappa ^{1,j}_i := \delta _{i,j}$ and $\kappa ^{2,j}_i := 2\delta _{i,j}$ for each $j\in \{1,\ldots ,n\}$, using the Kronecker $\delta$ notation; and $\mathcal {H}^q_{f,Y}$ is the multivariate polynomial containing the same terms as $\mathcal {P}^q_{f,Y}$ apart from those indexed by $\kappa ^1_j$ and $\kappa ^2_j$. Then, a similar rearrangement to the one proposed in [33] can be obtained by using the property that $T_2(\xi )=2T_1(\xi )^2-1$,

$$\begin{aligned} \mathcal {P}^q_{f,Y}({\xi })&= \sum _{j=1}^{n} \left[ 2a^q_{Y,\kappa ^{2,j}} \left( T_1(\xi _j)+\frac{a^q_{Y,\kappa ^{1,j}}}{4a^q_{Y,\kappa ^{2,j}}} \right) ^2 - a^q_{Y,\kappa ^{2,j}} - \frac{\left( a^q_{Y,\kappa ^{1,j}}\right) ^2}{8a^q_{Y,\kappa ^{2,j}}} \right] + \mathcal {H}^q_{f,Y}(\xi ). \end{aligned}$$

This way, an interval enclosure is obtained as

$$\begin{aligned} \left[ \mathcal {P}^q_{f,Y}\right] = \sum _{j=1}^{n} \left[ 2a^q_{Y,\kappa ^{2,j}} \left( [-1,1]+\frac{a^q_{Y,\kappa ^{1,j}}}{4a^q_{Y,\kappa ^{2,j}}} \right) ^2 - a^q_{Y,\kappa ^{2,j}} - \frac{\left( a^q_{Y,\kappa ^{1,j}}\right) ^2}{8a^q_{Y,\kappa ^{2,j}}} \right] + \left[ \mathcal {H}^q_{f,Y}\right] , \end{aligned}$$

with $[\mathcal {H}^q_{f,Y}]$ computed directly from (30). This bounder may only be considered when $|a^q_{Y,\kappa ^{2,j}}|\ge \epsilon$, where $\epsilon$ is a small positive number; otherwise, separate bounding of the linear and quadratic terms for the corresponding component *j* as in (30) may be used.

#### Bounding method of Bernstein

The Bernstein algorithm is a well-established tool for computing bounds on the range of multivariate polynomials over a box; see, e.g., [20, 30] and references therein. The procedure involves rewriting the multivariate polynomial from Chebyshev form into Bernstein form:

31 $$\begin{aligned} \forall \xi \in [-1,1]^n, \quad \mathcal {P}^q_{f,Y}(\xi ) = \sum _{\kappa \in \{0,\ldots ,r\}^{n}} b^q_{Y,\kappa } \prod _{i=1}^{n} B^r_{\kappa _i}\left( \frac{\xi _i+1}{2}\right) , \end{aligned}$$

with $r\ge q$, and where $B^r_i$, with $i\le r$, denotes the *i*th Bernstein polynomial of order *r* on [0, 1], given by

$$\begin{aligned} B_i^r(z) = \left( {\begin{array}{c}r\\ i\end{array}}\right) z^i(1-z)^{k-i}. \end{aligned}$$

In particular, the Bernstein coefficient $b^q_{Y,\kappa }$ in (31) can be expressed in terms of the Chebyshev coefficients $a^q_{Y,\kappa }$ as follows [48]

$$\begin{aligned} \forall \kappa \in \{0,\ldots ,r\}^{n}, \quad b^q_{Y,\kappa }&= \sum _{|\nu |\le q} a^q_{Y,\nu } \prod _{i=1}^{n} M_{\kappa _i,\gamma _i}\\ \text {with:} \quad M_{k,j}&:= \left( {\begin{array}{c}r\\ k\end{array}}\right) ^{-1} \sum _{i=\max \{0,j+k-r\}}^{\min \{j,k\}} (-1)^{j-i} \left( {\begin{array}{c}2j\\ 2i\end{array}}\right) \left( {\begin{array}{c}r-j\\ k-i\end{array}}\right) . \end{aligned}$$

This transformation turns out to be particularly well-conditioned from a numerical stability standpoint [48]. At this point, an interval enclosure of $\mathcal {P}^q_{f,Y}$ on $[-1,1]^n$ is simply obtained as

$$\begin{aligned} \left[ \mathcal {P}^q_{f,Y}\right] = \left[ \min _{\nu \in \{0,\ldots ,r\}^{n}} b^q_{Y,\nu }, \max _{\nu \in \{0,\ldots ,r\}^{n}} b^q_{Y,\nu } \right] . \end{aligned}$$

The main advantage of this approach is that the foregoing enclosure converges to the actual polynomial range in the Hausdorff sense as the order *r* increases [20]. Moreover, it is possible to bound the actual under- or over-estimation for a given order $r\ge q$.

## Convergence analysis

This section presents an analysis of the local convergence rate of Chebyshev models constructed from the application of the arithmetic operations described in Sect. 3. The global convergence of this arithmetic as the polynomial expansion order *q* increases is also discussed at the end of the section.

### Definition 2

Let the function $f:Z\rightarrow \mathbb {R}$ be defined on $Z\subseteq \mathbb {R}^n$. The scheme $(\mathcal {P}_{f,Y}^{q},\mathcal {R}_{f,Y}^{q})_{Y\subset Z}$ of *q*th-order Chebyshev models for *f*, with

$$\begin{aligned} \mathcal {P}_{f,Y}^{q}(\xi ) := \sum _{|\kappa |\le q} a^{q}_{Y,\kappa } T_\kappa (\xi ), \end{aligned}$$

is said to have *local convergence order r on Z* if

32 $$\begin{aligned} \forall \kappa \in \mathbb {N}^n\quad \text {with}\,|\kappa |\le q, \quad a^{q}_{Y,\kappa }&= \mathcal O\left( ({{\mathrm{rad\,}}}Y)^{\min \{|\kappa |,r\}}\right) \end{aligned}$$

33 $$\begin{aligned} \text {and}\quad {{\mathrm{rad\,}}}\mathcal {R}_{f,Y}^{q}&= \mathcal O\left( ({{\mathrm{rad\,}}}Y)^{r}\right) , \end{aligned}$$

for all interval vectors $Y\subset Z$ with sufficiently small ${{\mathrm{rad\,}}}Y$.

Notice the extra conditions (32) imposed on the coefficients of the polynomial approximant in the previous definition. While these conditions are not strictly necessary to establish local convergence, the propagation of (local) convergence order for the polynomial coefficients through binary sum, binary product and univariate outer-composition operations provides insight into the propagation of the convergence order for the remainder term in the following analysis.

As far as initialization is concerned, it is clear that a scheme of *q*th-order Chebyshev models $(\mathcal {P}_{z_i,Y}^{q},\mathcal {R}_{z_i,Y}^{q})_{Y\subset Z}$ for the variable $z_i\in Z_i$ with $i\in \{1,\ldots ,n\}$, as given by (11) and (12), has local convergence order 1 if $q=0$, and infinite order if $q\ge 1$. In both cases, the schemes of *q*th-order Chebyshev models for the variables thus have convergence order no less than $q+1$. Regarding the range bounders introduced in Sect. 3.5, the convergence condition (32) imposes that ${{\mathrm{rad\,}}}([\mathcal {P}_{f,Y}^{q}]+\mathcal {R}_{f,Y}^{q})=\mathcal O({{\mathrm{rad\,}}}Y)$ whenever the scheme $(\mathcal {P}_{f,Y}^{q},\mathcal {R}_{f,Y}^{q})_{Y\subset Z}$ has convergence order $r\ge 1$.

Next, we investigate the local convergence order of schemes of Chebyshev models as propagated through factorable functions, with the corresponding binary sum, binary product and univariate outer-composition operations derived in Sects. 3.2–3.4. With regards to binary sum operations given by (13)–(14), the sum of two schemes of *q*th-order Chebyshev models trivially preserves the least convergence order of the operands, be they smaller than, equal to, or larger than $q+1$. In particular, the addition/subtraction of two schemes with local convergence order $q+1$ is itself a scheme of order $q+1$. The propagation of convergence orders through binary product and univariate outer-composition operations is somewhat more involved, and detailed in Sects. 4.1 and 4.2.

### Local convergence rate of binary product operations

Adopting the notation introduced in Sect. 3.3, we assume here that the schemes of *q*th-order Chebyshev models $(\mathcal {P}_{f_1,Y}^{q},\mathcal {R}_{f_1,Y}^{q})_{Y\subset Z}$ and $(\mathcal {P}_{f_2,Y}^{q},\mathcal {R}_{f_2,Y}^{q})_{Y\subset Z}$ have local convergence orders $r_1\ge 1$ and $r_2\ge 1$ on *Z*, respectively.

The following lemma is instrumental to prove convergence of the Chebyshev coefficients in the product scheme:

#### Lemma 2

For any $q\ge 0$, any $\kappa \in \mathbb {N}^q$, and any pair $(\lambda ,\mu )\in \mathbb {P}_q(\kappa )$, we have $|\lambda +\mu |\ge |\kappa |$.

#### Proof

By construction of $\mathbb {P}_q(\kappa )$ in (15), we either have $\lambda _i+\mu _i=\kappa _i$ or $|\lambda _i-\mu _i|=\kappa _i$, for each $i\in \{1,\ldots ,n\}$. Therefore, the result follows by noting that $\lambda _i+\mu _i\ge |\lambda _i-\mu _i|$. $\square$

By the construction of the product polynomial in (16) and by Lemma 2, we have

34 $$\begin{aligned} \left| c^q_{Y,\kappa }\right| \le \sum _{(\lambda ,\mu )\in \mathbb {P}_q(\kappa )} \frac{\left| a^q_{Y,\lambda }\right| \left| b^q_{Y,\mu }\right| }{2^{N(\lambda ,\mu )}} = \mathcal O\left( ({{\mathrm{rad\,}}}Y)^{\min \{r_1,r_2,|\kappa |\}}\right) , \end{aligned}$$

for every $\kappa \in \mathbb {N}^n$ with $|\kappa |\le 2q$. Therefore, the product rule (17) propagates the least convergence order of the operands through the polynomial coefficients, up to order $|\kappa |$. Moreover, we have $[\mathcal {P}_{f_1,Y}^{q}] = \mathcal O(1)$, $[\mathcal {P}_{f_2,Y}^{q}] = \mathcal O(1)$, and

$$\begin{aligned}&\sum _{q<|\kappa |\le 2q} \left| c^q_{Y,\kappa }\right| [-1,1] = \mathcal O\left( ({{\mathrm{rad\,}}}Y)^{\min \{r_1,r_2,q+1\}}\right) , \end{aligned}$$

so it follows from the remainder propagation rule (18) that

35 $$\begin{aligned} {{\mathrm{rad\,}}}\mathcal {R}_{f_1 f_2,Y}^{q}&= \mathcal O\left( ({{\mathrm{rad\,}}}Y)^{\min \left\{ r_1,r_2,q+1\right\} }\right) . \end{aligned}$$

Overall, the least convergence order of the operands thus propagates through the remainder, up to order $q+1$. In particular, the product scheme has local convergence order $q+1$ whenever both operands have convergence order $q+1$. These results are summarized in the following theorem:

#### Theorem 1

Let $(\mathcal {P}_{f_1,Y}^{q},\mathcal {R}_{f_1,Y}^{q})_{Y\subset Z}$ and $(\mathcal {P}_{f_2,Y}^{q},\mathcal {R}_{f_2,Y}^{q})_{Y\subset Z}$ be schemes of *q*th-order Chebyshev models, with local convergence orders $r_1\ge 1$ and $r_2\ge 1$, respectively, for the functions $f_1,f_2:Z\subseteq \mathbb {R}^n\rightarrow \mathbb {R}$. Then, the product scheme $(\mathcal {P}_{f_1 f_2,Y}^{q},\mathcal {R}_{f_1 f_2,Y}^{q})_{Y\subset Z}$ given by (17) and (18) has local convergence order $\min \{r_1,r_2,q+1\}$.

### Local convergence rate of univariate outer-composition operations

Using the notation introduced in Sect. 3.4, we assume that the outer functions $F:X\subseteq \mathbb {R}\rightarrow \mathbb {R}$ is *s*-times continuously differentiable, and we consider the scheme of *q*th-order Chebyshev models $(\mathcal {P}_{F,W}^q,\mathcal R_{F,W}^q)_{W\subset X}$, as constructed from (25), (26) [resp. from (26), (28)] if $s\ge q+1$; or otherwise constructed from (25), (27) [resp. from (28), (29)] if $F^{(s)}$ has a finite total variation on *X*.

#### Lemma 3

The scheme $(\mathcal {P}_{F,W}^q,\mathcal R_{F,W}^q)_{W\subset X}$ has local convergence order ${\min \{s,q+1\}}$ on *X*.

#### Proof

See “Appendix 2”. $$\square $$

To carry out the analysis, we also assume that the scheme of *q*th-order Chebyshev models $(\mathcal {P}_{f,Y}^{q},\mathcal {R}_{f,Y}^{q})_{Y\subset Z}$ for the inner functions $f:Z\subseteq \mathbb {R}^n\rightarrow \mathbb {R}$ has local convergence order $r\ge 1$. Notice that the Clenshaw summations (20)–(22) consists of binary sum and product operations only and is initialized with constants. However, by construction of $(\mathcal {P}_{\varPhi ,Y}^{q},\mathcal {R}_{\varPhi ,Y}^{q})_{Y\subset Z}$ in (19), we have

$$\begin{aligned}&a^q_{Y,0}=0,\qquad \forall \kappa \in \mathbb {N}^n\quad \text {with}\, 1\le |\kappa |\le q,\quad a^q_{Y,\kappa }=\mathcal O\left( ({{\mathrm{rad\,}}}Y)^{\min \{|\kappa |,r\}-1}\right) ,\\&\quad \quad \text {and}\quad {{\mathrm{rad\,}}}\mathcal {R}_{\varPhi ,Y}^{q}=\mathcal O\left( ({{\mathrm{rad\,}}}Y)^{r-1}\right) , \end{aligned}$$

where $a^q_{Y,\kappa }$ denote the coefficients of $\mathcal {P}_{\varPhi ,Y}^{q}$. Even though $(\mathcal {P}_{\varPhi ,Y}^{q},\mathcal {R}_{\varPhi ,Y}^{q})_{Y\subset Z}$ has local convergence order $r-1$ only, the following lemma shows that the original order *r* of the inner operand nonetheless propagates through the scheme $(\mathcal {P}_{\mathcal {P}_F\circ \varPhi ,Y}^{q},\mathcal {R}_{\mathcal {P}_F\circ \varPhi ,Y}^{q})_{Y\subset Z}$, up to order $q+1$:

#### Lemma 4

The scheme $(\mathcal {P}_{\mathcal {P}_F\circ \varPhi ,Y}^{q},\mathcal {R}_{\mathcal {P}_F\circ \varPhi ,Y}^{q})_{Y\subset Z}$ generated through the Clenshaw recursion (20)–(22) satisfies

$$\begin{aligned} \forall \kappa \in \mathbb {N}^n\quad \text {with}\, |\kappa |\le q, \quad c^q_{Y,\kappa }&= \mathcal O\left( ({{\mathrm{rad\,}}}Y)^{\min \{|\kappa |,r,s\}}\right) ,\\ {{\mathrm{rad\,}}}\mathcal {R}_{\mathcal {P}_F\circ \varPhi ,Y}^{q}&= \mathcal O\left( ({{\mathrm{rad\,}}}Y)^{\min \{r,q+1\}}\right) , \end{aligned}$$

where $c^q_{Y,\kappa }$ denote the coefficients of $\mathcal {P}_{\mathcal {P}_F\circ \varPhi ,Y}^{q}$.

#### Proof

See “Appendix 3”. $$\square $$

A direct consequence of Lemmata 3 and 4 is that the remainder term $\mathcal {R}_{F\circ f,Y}^{q}$, as defined in (24), has convergence order $\min \{r,s,q+1\}$. Therefore, the convergence order of the inner operand may propagate through the remainder, up to order $q+1$, in a *q*th-order scheme of Chebyshev models. Moreover, if the outer operand is *s*-times continuously differentiable, the convergence order of the composition scheme may not be larger than *s*. In particular, the composition scheme has convergence order $q+1$ whenever the inner scheme has convergence order $q+1$ and the outer function is $q+1$-times continuously differentiable. These results are summarized in the following theorem:

#### Theorem 2

Let $(\mathcal {P}_{F,W}^{q},\mathcal {R}_{F,W}^{q})_{W\subset X}$ be a scheme of *q*th-order Chebyshev models for the *s*-times continuously-differentiable function $F:X\subseteq \mathbb {R}\rightarrow \mathbb {R}$, as constructed from (25), (26) [resp. from (26), (28)] if $s\ge q+1$; or otherwise constructed from (25), (27) [resp. from (28), (29)] if $F^{(s)}$ has a finite total variation on *X*. Let also $(\mathcal {P}_{f,Y}^{q},\mathcal {R}_{f,Y}^{q})_{Y\subset Z}$ be a scheme of *q*th-order Chebyshev models for the function $f:Z\subseteq \mathbb {R}^n\rightarrow \mathbb {R}$ with local convergence order $r\ge 1$. Then, the composition scheme $(\mathcal {P}_{F\circ f,Y}^{q},\mathcal {R}_{F\circ f,Y}^{q})_{Y\subset Z}$ given by (23) and (24) has convergence order $\min \{r,s,q+1\}$ on *Z*.

### Global convergence of Chebyshev model arithmetic

The previous convergence analysis investigates the rate at which the remainder bound shrinks as the domain *Y* of the variables is progressively reduced to a point, for a given expansion order *q*. This section discusses a different type of convergence, namely the property of the remainder bound to shrink to zero upon increasing the expansion order $q\rightarrow \infty$, for a fixed variable domain *Y*.

#### Definition 3

Let the function $f:Z\rightarrow \mathbb {R}$ be defined on $Z\subseteq \mathbb {R}^n$, and let $\mathbb {IR}^n\ni Y\subset Z$. The scheme $(\mathcal {P}_{f,Y}^{q},\mathcal {R}_{f,Y}^{q})_{q\ge 0}$ of *q*th-order Chebyshev models for *f*, with

$$\begin{aligned} \mathcal {P}_{f,Y}^{q}(\xi ) := \sum _{|\kappa |\le q} a^{q}_{Y,\kappa } T_\kappa (\xi ), \end{aligned}$$

is said to be *globally convergent on Y* if

36 $$\begin{aligned} \lim _{q\rightarrow \infty } \sum _{|\kappa |\le q} \left| a^{q}_{Y,\kappa }\right|&= \bar{A}_Y < \infty \quad \text {and}\quad \lim _{q\rightarrow \infty } \mathcal {R}_{f,Y}^{q} = \{0\}. \end{aligned}$$

As far as initialization is concerned, it is clear that a scheme of *q*th-order Chebyshev models $(\mathcal {P}_{y_i,Y}^{q},\mathcal {R}_{y_i,Y}^{q})_{q\ge 0}$ for the variable $y_i\in Y_i$ with $i\in \{1,\ldots ,n\}$, as given by (11) and (12), is globally convergent on *Y*. Regarding the range bounders introduced in Sect. 3.5, it is also worth noting that the sequence $\{{{\mathrm{rad\,}}}[\mathcal {P}_{f,Y}^{q}]\}_{q\ge 0}$ is bounded when (36) holds.

Next, we adopt the notation introduced in Sects. 3.2 and 3.3, and we assume that the schemes $(\mathcal {P}_{f_1,Y}^{q},\mathcal {R}_{f_1,Y}^{q})_{q\ge 0}$ and $(\mathcal {P}_{f_2,Y}^{q},\mathcal {R}_{f_2,Y}^{q})_{q\ge 0}$ are globally convergent on *Y*. The summation/subtraction scheme $(\mathcal {P}_{f_1\pm f_2,Y}^{q},\mathcal {R}_{f_1\pm f_2,Y}^{q})_{q\ge 0}$, as given in (14), is trivially globally convergent by the triangle inequality. Concerning the product operation in Sect. 3.3, we have from (16) that

$$\begin{aligned} \sum _{|\kappa |\le q} \left| c^{q}_{Y,\kappa }\right|&= \sum _{|\kappa |\le q} \left| \sum _{(\lambda ,\mu )\in \mathbb {P}_q(\kappa )} \frac{a^q_{Y,\lambda } b^q_{Y,\mu }}{2^{N(\lambda ,\mu )}}\right| \\&\le \sum _{|\kappa |\le q} \sum _{(\lambda ,\mu )\in \mathbb {P}_q(\kappa )} \frac{\left| a^q_{Y,\lambda }\right| \left| b^q_{Y,\mu }\right| }{2^{N(\lambda ,\mu )}}\\&= \sum _{|\lambda |\le q} \sum _{|\mu |\le q} \left| a^q_{Y,\lambda }\right| \left| b^q_{Y,\mu }\right| , \end{aligned}$$

so

$$\begin{aligned} \lim _{q\rightarrow \infty } \sum _{|\kappa |\le q} \left| c^{q}_{Y,\kappa }\right| \le \lim _{q\rightarrow \infty } \sum _{|\lambda |\le q} \left| a^{q}_{Y,\lambda }\right| \sum _{|\mu |\le q}\left| b^{q}_{Y,\mu }\right| < \infty , \end{aligned}$$

and, in particular,

$$\begin{aligned} \lim _{q\rightarrow \infty } \sum _{q<|\kappa |\le 2q} \left| c^{q}_{Y,\kappa }\right| \le \lim _{q\rightarrow \infty }\sum _{|\kappa |> q} \left| c^{q}_{Y,\kappa }\right| = 0. \end{aligned}$$

It then follows from (18) that the product scheme $(\mathcal {P}_{f_1 f_2,Y}^{q},\mathcal {R}_{f_1 f_2,Y}^{q})_{q\ge 0}$ is globally convergent on *Y*, as long as the sequences $\{{{\mathrm{rad\,}}}[\mathcal {P}_{f_1,Y}^{q}]\}_{q\ge 0}$ and $\{{{\mathrm{rad\,}}}[\mathcal {P}_{f_2,Y}^{q}]\}_{q\ge 0}$ are bounded. These considerations are summarized in the following theorem:

#### Theorem 3

Let $(\mathcal {P}_{f_1,Y}^{q},\mathcal {R}_{f_1,Y}^{q})_{Y\subset Z}$ and $(\mathcal {P}_{f_2,Y}^{q},\mathcal {R}_{f_2,Y}^{q})_{Y\subset Z}$ be globally convergent schemes of Chebyshev models for the functions $f_1,f_2:Z\subseteq \mathbb {R}^n\rightarrow \mathbb {R}$, respectively. Then, the summation/subtraction scheme $(\mathcal {P}_{f_1\pm f_2,Y}^{q},\mathcal {R}_{f_1\pm f_2,Y}^{q})_{q\ge 0}$ given by (13) and (14) is globally convergent on *Y*; and so is the product scheme $(\mathcal {P}_{f_1 f_2,Y}^{q},\mathcal {R}_{f_1 f_2,Y}^{q})_{q\ge 0}$ given by (17) and (18) for the range bounding methods in Sect. 3.5.

In order to formally establish global convergence of the Chebyshev model arithmetic presented in Sect. 3, one also needs to establish sufficient conditions on both the inner and outer operands of a composition operation in order for the resulting composition scheme to be globally convergent. The development of such conditions calls for an analysis of the propagation of the global convergence property through the Clenshaw recurrence, for which results are currently lacking. The numerical case studies presented in the following sections suggest that global convergence might be guaranteed under mild regularity conditions nonetheless, and this will be the topic of further research.

## Numerical implementation and case studies

This section presents numerical case studies that illustrate the convergence properties of Chebyshev models, and makes comparisons with Taylor models in terms of tightness and computational effort. All the computations that led to these results are performed with the library MC++ (http://omega-icl.bitbucket.org/mcpp/), which features classes for the evaluation of factorable functions in Taylor and Chebyshev arithmetics (along with other arithmetics). Verified interval libraries, such as PROFIL (http://www.ti3.tu-harburg.de/) or FILIB++ (http://www2.math.uni-wuppertal.de/~xsc/), are used to perform the remainder interval computations, but any round-off caused by operations between the polynomial coefficients is not accounted for in the current implementation. We note that approaches to account for errors due to floating-point arithmetic are well-documented for Taylor models [36, 43], and the very same approaches may be used for Chebyshev models in order to arrive at a fully verified implementation.

MC++ implements the Chebyshev model arithmetic and range bounding operations described in Sect. 3, based on a dense representation of the multivariate polynomials. If not otherwise noted, Chebyshev models for the intrinsic functions are computed based on the Chebyshev interpolation polynomial. Moreover, the exact remainder bounding approach established in Sect. 3.4 is used for the intrinsic functions $\exp (x)$, $\log (x)$, $\sqrt{x}$, and $\frac{1}{x}$, and the default range bounder uses exact bounding of first- and diagonal second-order terms as described in Sect. 3.5. Another improvement involves intersecting the polynomial model bounds with those derived from simple interval analysis at each operation as a means of tightening the remainder bound, avoiding division by zero, etc; this extension is similar in essence to the mixed affine-arithmetic/interval-arithmetic model by Stolfi and Figueiredo [19].

**Fig. 1:**
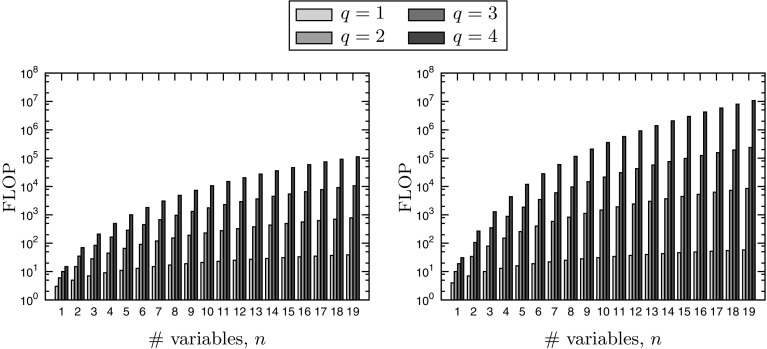
Number of FLOP for performing polynomial product in monomial basis (*left plot*) and in Chebyshev basis (*right plot*)

The most critical operation in an efficient implementation is multiplication of Chebyshev models, since it is used extensively in univariate outer-composition operations besides bivariate product operations. The approach used in MC++ involves constructing the index sets $\mathbb {P}_q(\kappa )$ given by (15), for all $\kappa \in \mathbb {N}^n$ with $|\kappa |\le q$, prior to propagating the Chebyshev models through a DAG of the factorable function. The number of floating-point operations (FLOP) required to compute the product polynomial $\mathcal {P}_{f_1 f_2,Y}^{q}$ in (17) is shown on the left plot of Fig. 1 for Chebyshev expansions of order $q=1,\ldots ,4$ and for functions with up to $n=19$ variables. Even for low expansion orders does performing polynomial products in Chebyshev basis result in an increase by 1 or 2 orders of magnitude in the number of FLOP, compared with monomial basis which is shown on the right plot. The better approximation capability of Chebyshev models may thus come at the price of a much higher computational burden compared with Taylor models, at least in a dense implementation. This refinement calls for the development of sparse implementations for both Taylor and Chebyshev model arithmetics in order to overcome this limitation and tackle larger-scale problems, a topic for future research.

### Example 1

**Fig. 2:**
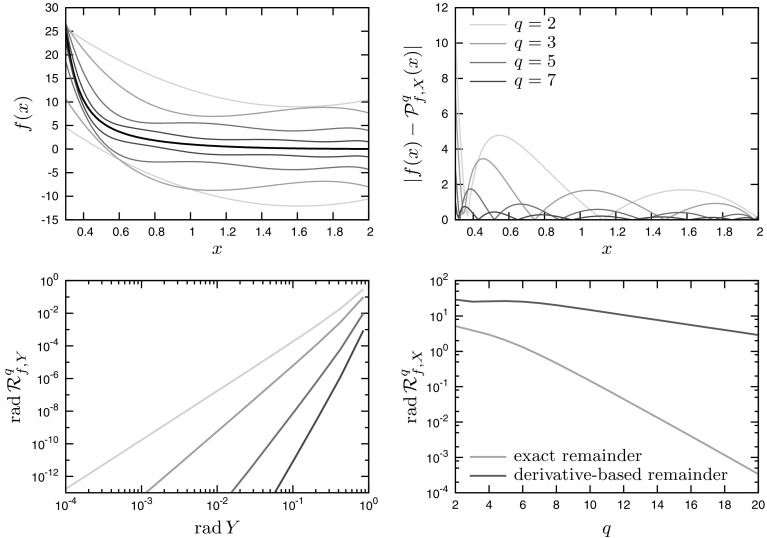
Application of Chebyshev models to Example 1. *Top-left plot* polynomial model enclosures of the function *f* (*black line*) for expansion orders $q=2,3,5,7$. *Top-right plot* approximation error for expansion orders $q=2,3,5,7$. *Bottom-left plot* local convergence analysis for a sequence of shrinking interval sets $Y\subset X$ around $x=1$. *Bottom-right plot* global convergence analysis on *X* as $q\rightarrow \infty$ with the exact remainder estimate compared with the high-order derivative-based remainder formula

We consider the univariate function *f* given by

$$\begin{aligned} f(x) = \exp \left( -x^2+\frac{1}{x}\right) , \end{aligned}$$

for the variable $x\in X:=[0.3,2]$. A number of Chebyshev model enclosures for various expansion orders *q* in the range 2-7 are shown on the top-left plot of Fig. 2, along with the corresponding approximation errors on the opposite plot. The scheme of Chebyshev models appears to be globally convergent as $q\rightarrow \infty$, both with the derivative-based remainder formula and with the exact remainder formula for the univariate outer-composition operations, as shown on the bottom-right plot of Fig. 2. However, the use of the exact remainder significantly tightens the bounds and improves the rate of convergence. In contrast, classical Taylor models fail to converge to the function as the order *q* is increased, despite the function *f* being analytic on the set *X*. This is because the variable range of interest is partially outside the radius of convergence for this function, which also means that the width of the interval remainder increases with *q*. Finally, the bottom-left plot on Fig. 2 shows that local convergence of *q*th-order Chebyshev model is of order $q+1$ around $x=1$. This observation is consistent with the analysis conducted in Sect. 4 since *f* is analytic.

**Fig. 3:**
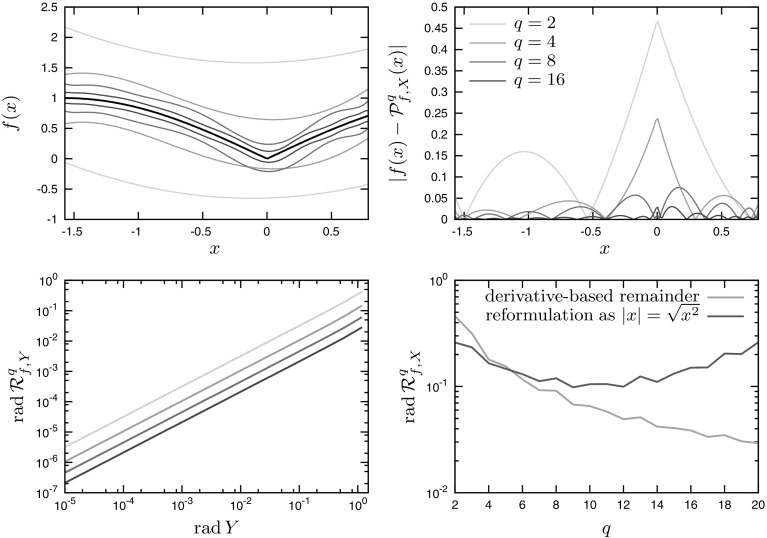
Application of Chebyshev models to Example 2. *Top-left plot* Polynomial model enclosures of the function *f* (*black line*) for expansion orders $q=2,4,8,16$. *Top-right plot* approximation error for expansion orders $q=2,4,8,16$. *Bottom-left plot* local convergence analysis for a sequence of shrinking interval sets $Y\subset X$ around $x=0$. *Bottom-right plot* global convergence analysis on *X* as $q\rightarrow \infty$ with the derivative-based remainder formula or the reformulation as $|x|=\sqrt{x^2}$

### Example 2

We consider the univariate function *f* given by

$$\begin{aligned} f(x) = \sin |x|, \end{aligned}$$

for the variable $x\in X:=\left[ -\frac{\pi }{2},\frac{\pi }{4}\right]$. Notice that classical Taylor models may not be used to bound the function *f* on *X* given the nonsmoothness at $x=0$. In contrast, Chebyshev models can be constructed since the univariate $\sin (x)$ is analytic in $\mathbb {R}$, whereas the derivative of |*x*| has total bounded variation on *X*. The top plots in Fig. 3 shows how the bounds improve with a larger expansion order *q*, up to $q=16$. It is evident that a much higher-order expansion is necessary in comparison with the smooth function in the previous example (Sect. 5.1), and that the approximation error is the largest close to $x=0$. In agreement with the theory in Sect. 4, the local convergence of any *q*th-order Chebyshev model at this point is of order 1, regardless of the expansion order $q \ge 0$; a prediction that is confirmed in the bottom-left plot of Fig. 3. But despite the nonsmoothness at $x=0$ and the linear local convergence around this point, Chebyshev models are found to be globally convergent on *X* as $q\rightarrow \infty$, when the remainder formula (29) is used for bounding the remainder of the univariate term |*x*| (bottom-right plot on Fig. 2).

Notice that this function may also be reformulated by substitution of $|x| = \sqrt{x^2}$, thus providing an alternative route to creating the Chebyshev model. This reformulation makes the use of the exact remainder estimate for the square-root term possible, which explains why tighter bounds for Chebyshev models of order $q=5$ or less are obtained here with the reformulation approach (bottom-right plot of Fig. 3). However, the reformulation also causes Chebyshev models to diverge on *X* as $q\rightarrow \infty$, a behavior attributed to the fact that the total variation of the square-root term is unbounded on intervals containing zero.

**Fig. 4:**
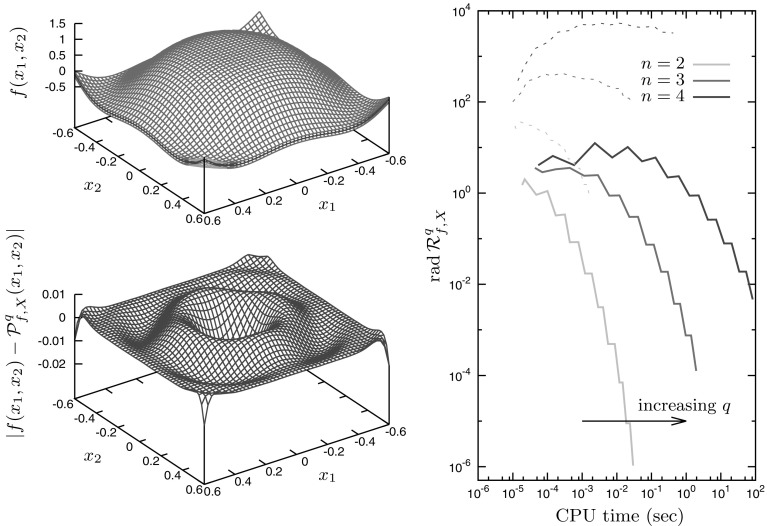
Application of Chebyshev models to Example 3. *Top-left plot* polynomial model enclosing the function *f* for $n=2$ with expansion orders $q=8$. *Bottom-left plot* corresponding approximation error for expansion orders $q=8$. *Right plot* computational effort and global convergence of the Chebyshev remainder ${{\mathrm{rad\,}}}\mathcal R^q_{f,X}$ with expansion orders $q=2,\ldots ,20$ for dimensions $n=2$ to 4 (*solid lines*), and comparison with their Taylor models counterparts (*dashed lines*)

### Example 3

As a final example, we consider the multivariate function *f* given by

$$\begin{aligned} f(x) = \exp \left( \sum _{i=1}^n x_i\right) \cos \left( 2\pi \sum _{i=1}^n x_i\right) , \end{aligned}$$

for the variable $x\in X := [-0.6,0.6]^n$, with the dimension *n* taken as 2, 3 or 4. The Chebyshev model enclosure and corresponding approximation error in the 2-variable case are presented on the left part of Fig. 4 for an expansion of order $q=8$, showing a tight approximation of the function. From the right plot of Fig. 4, it is seen that the computational burden increases with both the expansion order *q* and the dimension *n* of the problem. Moreover, computing Chebyshev models (solid lines) is more demanding than Taylor models (dashed lines), due to the extra complexity of the polynomial product in Chebyshev basis (see Fig. 1 above and related discussion). However, this extra complexity pays off in terms of the tightness of the resulting bounds. For $n=2$, one must consider expansions of order greater than $q=18$ in order for a Taylor model’s remainder bound to be as tight as those given by a second-order Chebyshev models only. For $n=3$ or $n=4$ likewise, although the Taylor models appear to be globally convergent on *X*, an expansion order far greater than $q=20$ would be necessary in order to be competitive with just second-order Chebyshev models. Overall, Chebyshev models thus outperform their Taylor counterparts. Finally, it is interesting to note that as the function *f* being even, odd ordered polynomial models prove to provide tighter bounds than even ones, thus explaining why there is sometimes little improvement (or even deterioration) in the bounds even though the computational time increases.

## Conclusions

This paper has formalized an arithmetic for the propagation of Chebyshev models through binary sums, binary products and univariate outer-composition operations in factorable functions. A simple method to calculate the exact remainder bounds for certain univariate terms, including $\exp (\cdot )$, $\log (\cdot )$, $\sqrt{\cdot }$ and $(\cdot )^{-1}$, is established. Adaptations of existing range bounders for Taylor models in order to use them with Chebyshev models are also discussed, which are a vital part of polynomial model arithmetic. The local convergence of Chebyshev models has been analyzed and proven to be equivalent to Taylor models, although the Chebyshev model remainder bounds are often found to be orders of magnitude better than their Taylor model counterparts over larger variable domains. This behavior is supported by the result that Chebyshev models are globally convergent through addition/subtraction and multiplication operations. The global convergence property is also conjectured for composition of Chebyshev models under mild conditions, although sufficient conditions are yet to be formally established. Such convergence properties have been illustrated through several numerical examples, based on an implementation in the authors’ in-house library MC++. These examples also illustrate some of the advantages of Chebyshev models over Taylor models, including being able to bound functions with points of non-smoothness or a divergent Taylor expansion. Even though Chebyshev models of equivalent order are computationally more expensive to create than their Taylor counterparts, mainly due to the much greater number of operations required for the binary product operation, they provide benefits by being significantly tighter. This may allow for a much lower order polynomial model to be used and creates a net benefit in terms of computational effort.

On the computational side, the current implementation creates a polynomial which is of the same order for all the variables and is stored using a dense representation. For many applications however, a large number of coefficients may be equal to zero, thereby calling for a sparse implementation of Chebyshev model arithmetic. Such an implementation would reduce both the computational time and the memory requirements. Another area of improvement would concern the binary product operation, whose complexity currently scales exponentially in the number of variables and the expansion order. Several studies have explored approaches to speeding up the multiplication of univariate and bivariate polynomials in Chebyshev basis (e.g., [2, 5, 22, 45]). Ways to use some of these developments for multivariate Chebyshev polynomials could be explored as part of future work. Binary product operations are also where much of the overestimation occurs, given that the polynomial part of the Chebyshev model product may not match the Chebyshev expansion of the product function. Instead, it might be possible to directly create multivariable Chebyshev approximations, for instance by following a similar approach as in chebfun2 [56]. This idea is also similar to the recent work on multivariate McCormick relaxations [59], where instead of decomposing the factorable function down to binary sums, products and univariate compositions, it would be possible to directly bound some multivariate terms.

## Appendix 1: Proof of Lemma 1

Since *F* in Lipschitz continuous on $[-1,1]$, it has a unique representation as an absolutely and uniformly convergent Chebyshev series, and we have

$$\begin{aligned} \forall \xi \in [-1,1], \quad F(\xi )-\mathcal {P}^q_F(\xi ) = \sum _{k=q+1}^\infty a_k T_k(\xi ), \end{aligned}$$

with the Chebyshev coefficients given by

37 $$\begin{aligned} a_k:=\frac{2}{\pi }\int _0^\pi f(\cos (\theta ))\cos (k\theta )d\theta . \end{aligned}$$

Observe that $\Vert F-\mathcal {P}^q_F\Vert = \sum \nolimits _{k=q+1}^\infty |a_k|$ under the following scenarios:

(i) the coefficients $a_k$ with $k\ge q+1$ are of the same sign, either non-negative or non-positive, in which case $|F(1)-\mathcal {P}^q_F(1)| = \sum \nolimits _{k=q+1}^\infty |a_k|$ by the property that $T_k(1)=1, \forall k\ge 0$;
(ii) the coefficients $a_k$ with $k\ge q+1$ alternate sign, either non-negative for even coefficients and non-positive for odd coefficients or vice-versa, in which case $|F(-1)-\mathcal {P}^q_F(-1)| = \sum \nolimits _{k=q+1}^\infty |a_k|$ by the property that $T_k(-1)=(-1)^k, \forall k\ge 0$.

Without loss of generality, we shall prove that case (i) holds with non-negative Chebyshev coefficients $a_k$ when *F* has an analytic extension on the closed unit disk and all its successive derivatives are nonnegative on $[-1,1]$; the other three cases follow readily by symmetry, considering $-F(\xi )$, $F(-\xi )$ or $-F(-\xi )$.

Under the assumption that *F* has an analytic extension on the closed unit disk, its Taylor series at any point $\bar{\xi }\in [-1,1]$ converges to *F* on $[-1,1]$. At $\bar{\xi }=0$ in particular, we have

38 $$\begin{aligned} \forall \xi \in [-1,1], \quad F(\xi ) = \sum _{k=0}^\infty \frac{F^{(k)}(0)}{k!}\xi ^k = \sum _{k=0}^\infty a_k T_k(\xi ^k). \end{aligned}$$

Recall that any polynomial $\mathcal {P}^q$ of order $q\ge 1$, given in monomial basis by $\mathcal {P}^q(\xi ):=\sum \nolimits _{k=0}^q \alpha _k\xi ^k$, has a unique representation in Chebyshev basis as $\mathcal {P}^q(\xi )=\sum \nolimits _{k=0}^q \beta _kT_k(\xi )$ with

$$\begin{aligned} \forall k\in \{1,\ldots , q\}, \quad \beta _k := \sum _{i=1}^q \frac{\alpha _i}{2^i} \sum _{j=0}^i \left( {\begin{array}{c}i\\ j\end{array}}\right) \delta _{k,|i-2j|}. \end{aligned}$$

By passing to the limit $q\rightarrow \infty$, we obtain from (38) that

39 $$\begin{aligned} \forall k\ge 1, \quad a_k := \sum _{i=1}^\infty \frac{F^{(i)}(0)}{2^i i!} \sum _{j=0}^i \left( {\begin{array}{c}i\\ j\end{array}}\right) \delta _{k,|i-2j|}. \end{aligned}$$

If follows from the nonnegativity of all the successive derivatives $F^{(i)}$ that $a_k\ge 0$ for every $k\ge 1$.

Regarding Chebyshev interpolation, on the other hand, we have

$$\begin{aligned} \forall \xi \in [-1,1], \quad F(\xi )-\widehat{\mathcal {P}}^q_F(\xi ) = \sum _{k=0}^q \left( a_k-\widehat{a}_k\right) T_k(\xi ) + \sum _{k=q+1}^\infty a_k T_k(\xi ), \end{aligned}$$

with the coefficients $\widehat{a}_k$ given by

40 $$\begin{aligned} \widehat{a}_k := \frac{2}{q+1}\sum _{j=0}^q F(\zeta _j) T_k(\zeta _j) \quad \text {with} \zeta _j := \cos \left( \frac{\pi }{2}\frac{j+1}{q+1}\right) . \end{aligned}$$

Like previously, we shall prove that

$$\begin{aligned} \left\| F -\widehat{\mathcal {P}}^q_F\right\| = \left| F(1)-\widehat{\mathcal {P}}^q_F(1)\right| = \pm \left( a_0-\widehat{a}_0\right) + \sum _{k=1}^q \left( a_k-\widehat{a}_k\right) + \sum _{k=q+1}^\infty a_k \end{aligned}$$

in the case that *F* has an analytic extension on the closed unit disk and all its successive derivatives are nonnegative on $[-1,1]$—the other cases following by symmetry. Since $a_k\ge 0$ for every $k\ge 1$ under these assumptions, it is sufficient to prove that $a_k \ge \widehat{a}_k$ for each $k=1,\ldots , q$. By the aliasing property of the Chebyshev polynomials [37], we have

41 $$\begin{aligned} \forall k\in \{0,\ldots ,q\},\quad \widehat{a}_k = a_k - a_{2q+2-k} - a_{2q+2+k} + a_{4q+4-k} + a_{4q+4+k} - \cdots \end{aligned}$$

and, therefore, the result follows by showing that $a_k\ge a_{k+2}$ for every $k\ge 1$. Considering the case of odd coefficients first, (39) gives

$$\begin{aligned} a_{2k-1}&= \sum _{i=k}^\infty \frac{F^{(2i-1)}(0)}{2^{2i-2} (2i-1)!} \left( {\begin{array}{c}2i-1\\ i-k\end{array}}\right) \\&= \frac{F^{(2k-1)}(0)}{2^{2k-2} (2k-1)!} + \sum _{i=k+1}^\infty \frac{F^{(2i-1)}(0)}{2^{2i-2} (2i-1)!} \left( {\begin{array}{c}2i-1\\ i-k\end{array}}\right) \\&= \frac{F^{(2k-1)}(0)}{2^{2k-2} (2k-1)!} + \sum _{i=k+1}^\infty \frac{F^{(2i-1)}(0)}{2^{2i-2} (2i-1)!} \left( {\begin{array}{c}2i-1\\ i-k-1\end{array}}\right) \frac{i+k}{i-k}\\&\ge \frac{F^{(2k-1)}(0)}{2^{2k-2} (2k-1)!} + a_{2k+1}, \end{aligned}$$

and a similar result holds for even coefficients. $\square$

## Appendix 2: Proof of Lemma 3

Let the Chebyshev coefficients of the function *F* on the subset $W\subset X$ be denoted as

$$\begin{aligned} \forall k\ge 1, \quad a_{W,k}:=\frac{2}{\pi }\int _{-1}^{1} \frac{F({{\mathrm{mid\,}}}W+\xi {{\mathrm{rad\,}}}W)T_k(\xi )}{\sqrt{1-\xi ^2}}d\xi . \end{aligned}$$

Since *F* is *s*-times continuously differentiable, Taylor’s theorem asserts that, for all $\xi \in [-1,1]$, there exists $\zeta _\xi \in [-1,1]$ such that

$$\begin{aligned} F({{\mathrm{mid\,}}}W+\xi {{\mathrm{rad\,}}}W) = \sum _{i=0}^{s-1} \frac{F^{(i)}({{\mathrm{mid\,}}}W)}{i!}(\xi {{\mathrm{rad\,}}}W)^i + \frac{F^{(s)}(\zeta _\xi )}{s!}(\xi {{\mathrm{rad\,}}}W)^s. \end{aligned}$$

Therefore, letting $M_i := \Vert F^{(i)}\Vert _X$ for $0\le i\le s$, we have

$$\begin{aligned} |a_{W,k}|&\le \sum _{i=0}^{s-1} \frac{2({{\mathrm{rad\,}}}W)^i M_i}{\pi \,i!} \left| \int _{-1}^{1} \frac{\xi ^i T_k(\xi )}{\sqrt{1-\xi ^2}}d\xi \right| + \frac{2({{\mathrm{rad\,}}}W)^s M_s}{\pi \,s!} \int _{-1}^{1} \frac{\left| \xi ^s T_k(\xi )\right| }{\sqrt{1-\xi ^2}}d\xi . \end{aligned}$$

Since $\int _{-1}^{1} \frac{\xi ^i T_k(\xi )}{\sqrt{1-\xi ^2}}d\xi =0$ for all $i<k$, $|\int _{-1}^{1} \frac{\xi ^i T_k(\xi )}{\sqrt{1-\xi ^2}}d\xi |\le \pi$ for all $i\ge 0$, and $\int _{-1}^{1} \frac{|\xi ^s T_k(\xi )|}{\sqrt{1-\xi ^2}}d\xi \le \pi$, we obtain

$$\begin{aligned} \forall k\ge 1, \quad |a_{W,k}|&\le ({{\mathrm{rad\,}}}W)^k \sum _{i=k}^{s} \frac{2({{\mathrm{rad\,}}}W)^{i-k}M_i}{i!} + ({{\mathrm{rad\,}}}W)^s \frac{2M_s}{s!}\\&= \mathcal O\left( ({{\mathrm{rad\,}}}W)^{\min \{k,s\}} \right) . \end{aligned}$$

Using Chebyshev interpolation likewise, a direct consequence of the aliasing property (41) is that

$$\begin{aligned} \forall k\in \{1,\ldots ,q\}, \quad \widehat{a}_{W,k}&= \mathcal O\left( ({{\mathrm{rad\,}}}W)^{\min \{k,s\}} \right) . \end{aligned}$$

The result of the lemma follows by noting that ${{\mathrm{rad\,}}}\mathcal {R}_{F,W}^{q} = \mathcal O(({{\mathrm{rad\,}}}W)^{\min \{s,q+1\}})$ by (26). $\square$

## Appendix 3: Proof of Lemma 4

We start by showing, using finite recursion on $k=q+2,q+1,\ldots , 1$, that the intermediate schemes $(\mathcal {P}_{\beta _k,Y}^{q},\mathcal {R}_{\beta _k,Y}^{q})_{Y\subset Z}$ all satisfy

42 $$\begin{aligned} \forall \kappa \in \mathbb {N}^n, \quad b^q_{k,Y,\kappa }&= \left\{ \begin{array}{ll} \mathcal O\left( ({{\mathrm{rad\,}}}Y)^{\min \{|\kappa |+k,r,s\}}\right) &{}\quad \text {if }\,0\le |\kappa |\le q-k\\ 0 &{}\quad \text {otherwise}\end{array}\right. \end{aligned}$$

43 $$\begin{aligned} {{\mathrm{rad\,}}}\mathcal {R}_{\beta _k,Y}^{q}&= \mathcal O\left( ({{\mathrm{rad\,}}}Y)^{\min \{r,q+1\}}\right) , \end{aligned}$$

where $b^q_{k,Y,\kappa }$ denote the coefficients of $\mathcal {P}_{\beta _k,Y}^{q}$.

- This property is trivially satisfied for the schemes $(\mathcal {P}_{\beta _k,Y}^{q+1},\mathcal {R}_{\beta _k,Y}^{q+1})_{Y\subset Z}$ and $(\mathcal {P}_{\beta _k,Y}^{q+2},\mathcal {R}_{\beta _k,Y}^{q+2})_{Y\subset Z}$.
- Next, assume that the property holds for both $(\mathcal {P}_{\beta _k,Y}^{k+1},\mathcal {R}_{\beta _k,Y}^{k+1})_{Y\subset Z}$ and $(\mathcal {P}_{\beta _k,Y}^{k+2},\mathcal {R}_{\beta _k,Y}^{k+2})_{Y\subset Z}$, and consider the resulting scheme $(\mathcal {P}_{\beta _k,Y}^{k},\mathcal {R}_{\beta _k,Y}^{k})_{Y\subset Z}$ through (21). Since $[\mathcal {P}_{\varPhi ,Y}^{q}]=\mathcal O(1)$ and $[\mathcal {P}_{\beta _k,Y}^{k+1}]=\mathcal O(({{\mathrm{rad\,}}}Y)^{\min \{k+1,r,s\}})$, the remainder of the product scheme $(\mathcal {P}_{\varPhi ,Y}^{q},\mathcal {R}_{\varPhi ,Y}^{q}) (\mathcal {P}_{\beta _{k+1},Y}^{q},\mathcal {R}_{\beta _{k+1},Y}^{q})$ has order $\mathcal O(({{\mathrm{rad\,}}}Y)^{\min \{r,q+1\}})$, and so $\mathcal {R}_{\beta _k,Y}^{k}$ satisfies condition (43). Concerning the polynomial part, the effect of multiplying $\mathcal {P}_{\beta _{k+1},Y}^{q}$ with $\mathcal {P}_{\varPhi ,Y}^{q}$ is a reduction of the order of all the coefficients $b^q_{k+1,Y,\kappa }$ by one order. Moreover, subtracting $\mathcal {P}_{\beta _{k+2},Y}^{q}$ does not change the order of the coefficients and by Lemma 3 the lead coefficient $\varphi ^q_{B^q_{Y},k}$ has order $\mathcal O(({{\mathrm{rad\,}}}Y)^{\min \{k,s\}})$. Therefore, the coefficients $b^q_{k,Y,\kappa }$ satisfy condition (42), too.

The same reasoning applies in taking the recurrence one step further through (20), thereby giving the result. $\square$
